# Pediatric Vulvar Bleeding due to Leech Infestation: A Case Report

**Published:** 2018

**Authors:** Hajar ZIAEI HEZARJARIBI, Mahdi FAKHAR, Rezvan YALVEH, Sayed Abdollah MOUSAVI, Elham Sadat BANIMOSTAFAVI

**Affiliations:** 1. Molecular and Cell Biology Research Center, School of Medicine, Mazandaran University of Medical Sciences, Sari, Iran; 2. Student Research Committee, School of Medicine, Mazandaran University of Medical Sciences, Sari, Iran; 3. Surgery Ward, Booali Sina Hospital, Mazandaran University of Medical Sciences, Sari, Iran; 4. Dept. of Radiology, Imam Khomeini Hospital, Mazandaran University of Medical Sciences, Sari, Iran

**Keywords:** Children, Vulvar bleeding, Leech

## Abstract

Leech infestation most frequently occurs in upper body cavities of children including pharynx, nose and esophagus and, more rarely, the vagina and vulva. Here we describe a 6-yr-old girl with vulvar bleeding caused by leech infestation that referred to the Emergency unit of the Booali Sina Hospital in Sari, Iran in Sep 2015. She had a history of swimming in a pond prior to the occurrence. The leech infestation particularly vulvar involvement among young girls is extremely rare and yet neglected event in the world.

## Introduction

Leeches are bloodsucking worm-like that belong to the phylum Annelida, subclass Hirudinea. The leech can infect various parts of the human body and frequently found in ponds (with fresh and or marine water), swamps, and on wet vegetation in tropical forests (1). Leech infestation most frequently occurs in upper body cavities of children including pharynx, nose and esophagus and, more rarely, the vagina and/or the vulva. Female genitalia involvement is commonly found in adult after swimming in a pond or lake with freshwater in rural areas (2–4).

## Case report

**Fig. 1:**
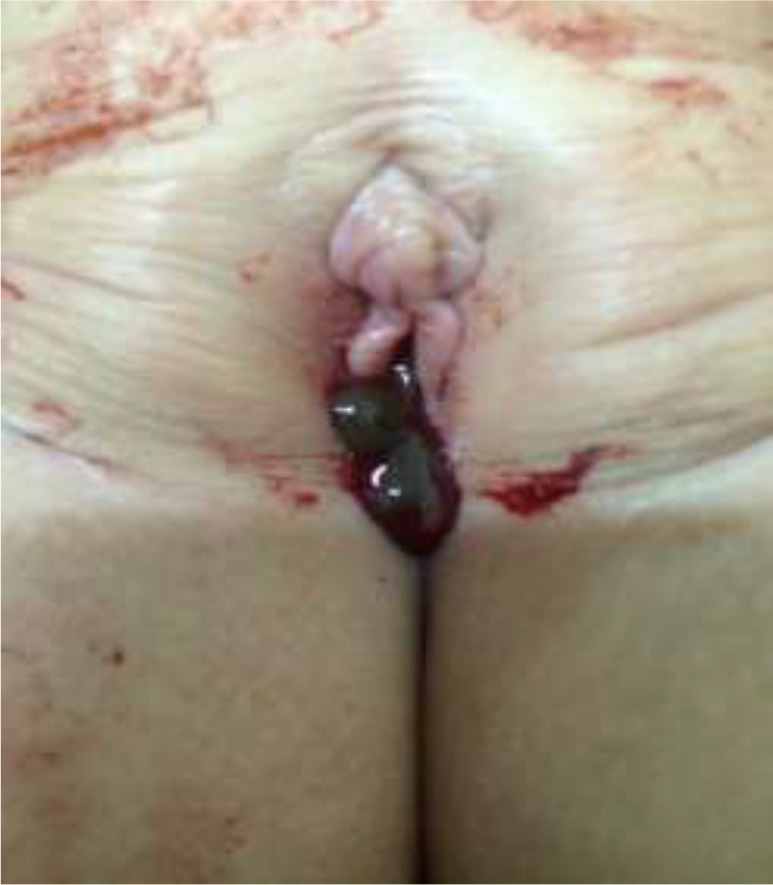
A leech on the patient’s vulva

A 6-yr-old girl who was traveling to rural area in the suburb area of Sari (Mazandaran Province, northern Iran) as tourist from Mashhad (Khorasan Razavi Province, eastern Iran) with vulvar bleeding, referred to the Emergency unit of the Booali Sina Hospital in Sari, northern Iran in Sep 2015,

She had such complaint for one week before admission and suffered from continuous moderate vulvar bleeding and dizziness. She denied having a history of trauma. The patient did not complain of any other symptoms. Through obtain history; her mother explained that who swim in a pond for about one hour. Clinical examination was achieved after her parents gave oral informed consent. Surprisingly, one leech about 5 cm in length was found in the minor labia of the vulva (Fig. 1).

No abnormality and trauma were seen and her hymen was intact. The leech was sent to the parasitology laboratory section and documented as leech belonging to Erpobdellidae family.

To improve the symptoms associated with vulvar bleeding, the leech was removed using forceps and washed twice the vulvar and vaginal cavity with normal saline and antibacterial solutions. The bleeding stopped 1 h later and the patient was discharged on the next day. She was followed up 2 d after removal of the leech; there was no symptom of infestation and bleeding.

## Discussion

Little is known regarding the incidence of leech infestation because the most frequent of the patient are referred to as outpatients. The majority of leeches (about 490 species) live in freshwater environments, while some species can be found in land (90 species) and marine (100 species) environments. Aquatic leeches can attach not only to the skin, but also to body cavity such as the mouth, throat, vagina, and urethra (5). About 29 leech species distributed in Iran, especially in the Caspian Sea areas such as Mazandaran Province (place of this report) with nine leech species belonging to Erpobdellidae family (6).

Genital leech infestation affecting vaginal and/or vulvar mucosa is very rare and mostly overlook neglected episode in children (2–4). Several cases have been reported regarding vaginal and or vulvular leech infestation in adult women particularly throughout summer season in wetness and tropical regions of the world including Iran (3,4,7–11), but vulvar involvement in young children, might not have been recorded formerly.

A careful history and gynecologic examination are very important in quick and early diagnosis of this less common infestation, particularly among young girls who have a history of swim in a pond. Our current report shows that leech infestation could have an impact on ecotourism. To prevent the occurrence of leech infestation, individuals should keep away from swimming in lakes or ponds (particularly in rural area), recreational and natural water resources.

## Conclusion

This report aware gynaecologist physicians that leech bite is part of the differential diagnosis among patients with poor personal hygienic who are suffering from bleeding in the genitalia and having a recent history of swimming in a pond or lake in wet rural areas where leech is common.
